# Evaluation of Permanent Deformation of CRM-Reinforced SMA and Its Correlation with Dynamic Stiffness and Dynamic Creep

**DOI:** 10.1155/2013/981637

**Published:** 2013-11-05

**Authors:** Nuha Salim Mashaan, Mohamed Rehan Karim

**Affiliations:** Center for Transportation Research, Faculty of Engineering, University of Malaya, 50603 Kuala Lumpur, Malaysia

## Abstract

Today, rapid economic and industrial growth generates increasing amounts of waste materials such as waste tyre rubber. Attempts to inspire a green technology which is more environmentally friendly that can produce economic value are a major consideration in the utilization of waste materials. The aim of this study is to evaluate the effect of waste tyre rubber (crumb rubber modifier (CRM)), in stone mastic asphalt (SMA 20) performance. The virgin bitumen (80/100) penetration grade was used, modified with crumb rubber at four different modification levels, namely, 6%, 12%, 16%, and 20% by weight of the bitumen. The testing undertaken on the asphalt mix comprises the indirect tensile (dynamic stiffness), dynamic creep, and wheel tracking tests. By the experimentation, the appropriate amount of CRM was found to be 16% by weight of bitumen. The results show that the addition of CRM into the mixture has an obvious significant effect on the performance properties of SMA which could improve the mixture's resistance against permanent deformation. Further, higher correlation coefficient was obtained between the rut depth and permanent strain as compared to resilient modulus; thus dynamic creep test might be a more reliable test in evaluating the rut resistance of asphalt mixture.

## 1. Introduction 

The growth of vehicle production year by year has generated wasted tyres. Due to limitation of disposal area and environmental problem, the recycling of these vehicles tyres as industrial wastes has been encouraged, and the production of rubber crumbs from then has found to be suitable for use as modifier into bitumen. Generally, there are two main factors which affect the performances of asphalt mixture which are the selected binder and mix composition; hence, highway construction engineers must consider the primary user's requirements of safety and economy. Therefore, highway construction designers should take into account three essential requirements which include environmental factors, traffic flow, and asphalt mixtures materials [1]. The asphaltic concrete wearing course which is used in the construction of flexible pavement derives its mechanical strength from the bonding of aggregates, sand, bitumen, and filler mortar. The performance of the mix will be strongly influenced by aggregate grading and the quantity and rheology of the bitumen in the mixture. Currently, Malaysia's road structures have deteriorated more rapidly due to increases in service traffic density, axle loading, and low maintenance services. The cause of damage to road surfacing is quite often traced to the adhesion failure. The weather condition in Malaysia leads to variation of temperature of about 55°C at the surface to 25°C at the subgrade during hot days. To minimise the damage of pavement surface and increase durability of flexible pavement, the conventional bitumen needs to be improved with regard to performance-related properties, such as resistance to permanent deformation (rutting). The modification of bituminous binder has been explored over the past years in order to improve road pavement performance properties [1–3]. Malaysia's production of scrap tyres is about 10 million pieces per annum, and unfortunately they are being disposed, in an environmentally unfriendly manner. Even though using commercial polymer offers the possibility to produce mixtures that can resist both rutting and cracking, it is costly. Thus, using waste materials such as crumb rubber is a good alternative and inexpensive [2]. 

Recycled waste tyre rubber is used as reclaimed rubber and referred to as crumb rubber. Tyre rubber is a blend of synthetic rubber, natural rubber, carbon black, antioxidants, fillers, and extender type of oils which are soluble in hot paving grade. Since the 1960s, the use of rubberised bitumen binder in road materials applications has gained increased interest in the paving industry of the USA [1–3]. In Malaysia, the use of rubber as an additive for road pavement construction started in the 1940s, but there has not been any official record of such practices. In 1993, rubberised asphalt road trial using waste gloves and natural rubber latex was carried out in Negeri Sembilan, Malaysia [4]. 

### 1.1. Study Aim and Objectives

Scrap tyres lead to serious disposal problems. However, the use of scrap tyres in asphalt pavements in the form of fillers/additives could minimise environmental pollution and maximise natural resource conservation. There are two major approaches to resolve the wastage of rubber and disposal of scrap tyres which are

- to reduce and reuse used and waste rubber;
- to reclaim raw resource of rubber.

The current study, therefore, attempts to evaluate the effect of waste tyre rubber (crumb rubber modifier (CRM)) obtained from vehicles waste tyres, in SMA 20S performance. The primary objectives are (1) to determine and compare some fundamental mix properties such as the resilient modulus and creep of the stone mastic asphalt (SMA) mixture as a result of the incorporation of crumb rubber; (2) to explore the rutting performance of SMA reinforced with crumb rubber; (3) to evaluate the relationship between the laboratory test parameters with respect to the rutting performance of SMA mixture reinforced with crumb rubber. 

## 2. Background

Road pavements are exposed to various traffic loads, changeable climatic cycles, and different soil characteristics of roadbed, which may result in distortion of pavement layers. These distortions may take the form of cracks, deformations, deterioration, and, failure and are located underneath the wheel tracks especially where the soil bearing capacity has been weakened during highly varied climatic temperatures. Excessive permanent deformation such as rutting is generally considered as the main and most harmful form of deterioration experienced; therefore special consideration is given to it in this section.

### 2.1. Rutting and Rubberised Asphalt

Rutting is known as longitudinal depressions, which follow the line of the wheel paths. The rutting of pavement deterioration is a major problem on heavily travelled flexible pavements specifically on climbing lanes. It is caused by permanent deformation due to traffic loading in one or more pavement layers. It is generally irregular and leads to a decrease in riding quality. This deterioration may occur due to lateral plastic deformations especially in high temperature in unstable wearing course or subgrade soil [5]. Field studies indicated that the rutting is usually influenced by the use of excessive binder content and improper aggregate gradation [6, 7]. This excessive binder essentially results in low air void and causes a loss of mechanical friction in the mineral skeleton and eventually leads to a greater level of plastic flow in bituminous pavement matrix [7]. Varied temperature, adhesion of bitumen with aggregate, speed of vehicle, amount and distribution of traffic, and surface contour are important factors to create rutting in pavements. There are various laboratory methods for studying distortion or rutting. The wheel tracking test appears to be the most suitable in stimulating the field conditions as closely as possible. The test was conducted for 24 hours in temperature-controlled cabinet at 60°C. From the indents made on the slab, the depth of tracking was recorded at the midpoint of its length. After about 6 hours, a steady state of tracking was observed. From the deformation/time curve, the rate of increase in track depth is determined in mm per hour once the steady state is reached [7]. According to Shih et al. [8], addition of crumb rubber and SBR increases the rutting resistance of asphalt paving mixtures. The results from laboratory study showed that the CR-modified and SBR-modified asphalt had higher stiffness at 60°C than the modified mixtures. The modified asphalt mixtures also had higher gyratory shear strengths and lower rut depths in the Loaded Wheel tests than the unmodified mixtures.

### 2.2. Resilient Modulus and Rubberised Asphalt

The dynamic stiffness or “resilient modulus” is a measure of the load-spreading ability of the bituminous layers; it controls the levels of the traffic-induced tensile strains at the underside of the lowest bituminous bound layer which are responsible for fatigue cracking, as well as the stresses and strains induced in the subgrade that can lead to plastic deformations [9]. The dynamic stiffness is computed by indirect tensile modulus test, which is a quick and nondestructive method. In general, the higher the stiffness, the better its resistance to permanent deformation and it improved the resistance to rutting [10]. Research on rubberised bitumen by Eaton et al. [11] shows that the resilient modulus increased or the mix behaved more stiffly (the mix got stronger) with a decrease in temperature; also, as the load time increased, the resilient modulus decreased or yielded more under a longer loading time.

Samsuri (1997) [10] reported that the elastic modulus of the samples produced using the preblended rubberised bitumen is approximately three times greater than that of the unmodified bitumen samples which is in accordance with the Marshall quotient results. Thus, the presence of binder modified by prelending with fine rubber powders improved the resistance to permanent deformation by nearly three times that of unmodified bituminous mixes. A study by Abdulla and Wai [12] reported that rubberised bituminous mixes show lower modulus compare to control mixes but at temperature above 25°C the rubberised bituminous mixes exhibit slightly higher modulus value. The laboratory results carried by Elcorp Technology Sdn. Bhd. showed that rubberised bituminous mixes have higher resilient modulus of about 20% than the conventional mix. Another study [13] found that at low temperature, rubber reduces the mix stiffness modulus; while at higher temperature, rubber increases the mix stiffness modulus at any given frequency. This makes rubberised bitumen a good binder to avoid rutting in hot climate and minimize brittle fracture at low temperature. Also, another study focused on the plastic deformation component of rutting. Georgia loaded wheel tester (GLWT) developed jointly by Georgia Tech and the Georgia Department of Transportation was used for this purpose. The measure of each beam was 7.5 cm deep, 13 cm wide and 38 cm long. The beams were conditioned for 24 hours at 40°C and then tested at 40°C. The results revealed that the conventional hot mix asphalt (HMA) mixes outperformed CRM mixes in resisting permanent deformation, both in the laboratory and the field. The Georgia loaded wheel tester results indicated that CRM mixes deform more and at a faster rate than conventional mixes [9].

### 2.3. Dynamic Creep and Rubberised Asphalt

Shear deformations resulting from high shear stresses in the upper portion of bituminous layer appear to be the primary cause of rutting in flexible pavements. Repeated applications of these stresses under conditions of comparatively low mix stiffness lead to the accumulation of permanent deformations at the pavement surface [9]. The creep test, carried out either in the static or dynamic mode of loading, is a useful test to be considered as part of a procedure for rutting evaluation for very slow-moving or static loads. The creep test gives the results which allow the characterization of the mixes in terms of their long-term deformation behaviour.

A research study [13] indicated that the addition of 4% rubber decreases the creep compliance of dense asphalt concrete by 42% at 45°C. Therefore, it is evident that asphalt concrete with 4% rubber shows fewer tendencies to strain during prolonged stress periods indicating its resistance to permanent deformation in hot climates, provided all structural parameters are adequate. It was reported that the total creep was higher for the rubber mixes, pointing out the benefits of their performance at lower temperatures, that is, greater elasticity and better resistance to thermal cracking [11].

## 3. Materials and Experimental Programme

### 3.1. Materials

**Figure 1:**
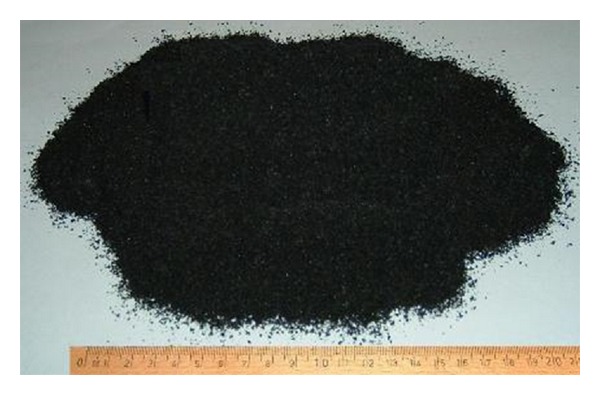
Crumb rubber size (0.6 mm).

For the purpose of this research investigation, bituminous binder of 80/100 penetration grade were used as illustrated in Table 1. The crushed granite aggregates supplied by the Kajang quarry (located near to Kuala Lumpur, Capital of Malaysia) was used throughout the study. The SMA 20 aggregate gradations were adopted, being characterised by 20 mm as shown in Table 2 [8]. In this study, in order to decrease segregation, fine crumb rubber size 30 (0.6 mm) from local supplier was selected, with specific gravity being equivalent to 1.161 as shown in Figure 1. CRM gradation is illustrated in Table 3.

### 3.2. Preparation of the SMA Mixture Samples

To incorporate rubber in bituminous mix, CRM is blended with the aggregate before adding the binder to the mixture. The specimens were prepared at optimum asphalt content (OBC) using Marshall method. Five various amounts of OBCs have been obtained for five various CRM contents, 5.20%, 6.30%, 6.42%, 6.50%, and 6.51% of OBC each for 0%, 6%, 12%, 16%, and 20% (all by weight of aggregate particles) of CRM content, respectively. In the current study 2% filler was used. For preparing SMA mixtures, 1100 g of mixed aggregate was placed in the oven at 160°C for 1.5 h. Bitumen was also heated at 120°C before mixing with aggregate particles. As the method of dry process, crumb rubber modifier was added directly to the mixture. Mixing temperature was kept constant at the temperature between 160 and 165°C. The mixture was transferred into a Marshall mould. The stainless steel thermometer was put in the centre of the mould and mixture was then ready for compaction at temperature of 160 ± 5°C. All samples were subjected to 50 blows of compaction by Marshall Hammer on each side of specimen at temperature of 150°C.

### 3.3. Testing Methods and Apparatus

The laboratory tests used to investigate and evaluate the performance properties of SMA bitumen mixture modified with CRM samples were indirect tensile, dynamic creep, and wheel tracking tests. 

#### 3.3.1. Indirect Tensile Modulus Test (Resilient Modulus)

This test covered the procedure for testing laboratory or filed recovered cores of bituminous mixtures to determine resilient modulus (MR) value using load indirect tensile test, under specified conditions of temperature, load, and load frequency. The test was conducted by applying compression loads with a prescribed sinusoidal waveform. The load was applied vertically in the vertical dimension plan of cylindrical specimen of bitumen sample. The resulting horizontal deformation of the specimen was measured with an assumed Poisson's ratio of 0.35 to calculate the resilient modulus values. The test was conducted at temperature of 40°C, pulse period equal to 1 second, and rise time equal to 70 ms. The universal materials testing apparatus (UMATTA) was used to carry out the indirect tensile test according to ASTM D4123.

#### 3.3.2. Dynamic Creep Test

This test simulates the passage of moving traffic loads on the pavement to study the permanent deformation characteristics of bituminous materials and its ability to resist the creep distress under repeated load. The test was conducted at temperature of 40°C for a period of 1 hour with loading stress of 100 kPa; the pulse period, pules width, terminal pulse, and conditioning stress count were at 2000 ms, 200 ms, 1800 counts, and 1 kPa, respectively, by using the UMATTA apparatus [9]. 

#### 3.3.3. Wheel tracking Test

This test determines the susceptibility of bituminous mixtures to deform plastically at high road temperatures under pressures similar to those experienced on the road. The susceptibility of bituminous materials to deform is assessed by the rut depth formed by repeated passes of loaded wheel at constant temperature. The purpose of this test is to determine the ability of asphalt mixture to resist rutting deformation. The test was conducted according to the British standard (BS 598 : 110).

## 4. Results and Discussion

### 4.1. Resilient Modulus

The modulus of resilience (MR) has emerged as the most convenient tool for measuring the stiffness modulus of asphalt mixture. This test is carried out under repeated loading at low stresses so that the response of the specimen tested remains elastic. The test was done according to ASTM D 4123 at 40°C. MR is the most fundamental variable in the approach to mechanistic design for the structure of road pavements. It reflects the pavement reaction against the dynamic stresses and corresponding strains [7].

**Figure 2:**
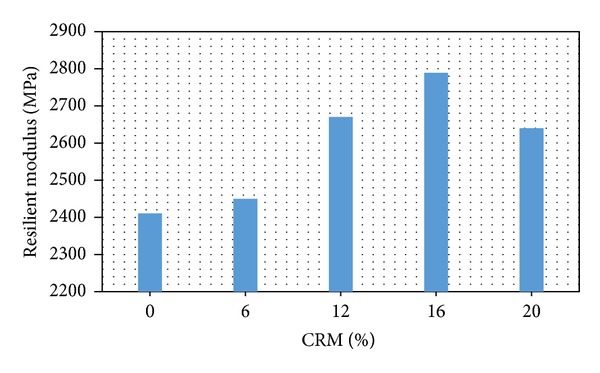
Resilient modulus (dynamic stiffness) versus CRM content.

 As Figure 2 displays, the value of the resilient modulus with different CRM-reinforced SMA in OBC is greater than the nonreinforced SMA (without CRM). This means that at optimum conditions the control samples (nonreinforced) have a bigger elastic deformation than the rubberised samples (CRM-reinforced SMAs) under dynamic traffic loading conditions. The results displayed an increase in resilient modulus as the crumb rubber content increases up to optimum value (16% CRM), then it decreases back with further increase of crumb rubber content. The reduction of the MR after adding more than 16% CRM can be related to excessive OBC content and depends mainly on the constant air void required. As revealed in Figure 2, there is a marked variation between the reinforced and nonreinforced samples in the stiffness modulus. In reinforced asphalt samples with CRM, the crumb rubber content absorbs a portion of bitumen resulting in the optimum binder percent to increase. As the crumb rubber content is increased, more bitumen is absorbed, which in turn increases the optimum binder content of the mix. It is evident that the stiffness modulus of reinforced asphalt samples is higher compared to the nonreinforced samples; however, the results of this research agrees the finding with of previous studies [14, 15].

### 4.2. Dynamic Creep Results

**Figure 3:**
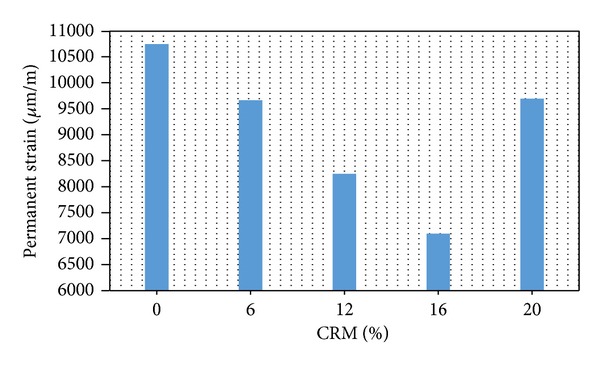
Permanent strain (dynamic creep) versus CRM content.

The dynamic creep test is used because it is ranked between the wheel tracking test and static creep and its ability to quantify the permanent deformation of the asphalt mix. The results obtained for dynamic creep test represented by the permanent strain for mixes at OBC with different CRM content at testing temperature of 40°C are given in Figure 3. The results obtained displayed the same trend as before, that is a decrease in strain values as the crumb rubber content increases and with further inclusion of rubber the strain value increases back. Crumb rubber content of 16% provided the lowest strain value. The addition of 12 and 16% CRM to the mix reduces the strain value significantly as compared to the control mix. The dynamic creep data suggests that at the optimum condition the addition of more than 16% CRM will cause minimal effect on resistance to permanent deformation. Also, following the trend of the plotted data as shown in Figure 3, it is expected that with further inclusion of CRM and beyond certain CRM content the strain value of the mixture will increase higher than with that of the non-reinforced mix causing a detrimental effect to the reinforced mix by reducing its resistance to permanent deformation. According to a study attributed to the effect of rubber content, enhancing the rubber content led to the increase in carbon black reacting with the natural rubber, which corresponded to the elastic part of the crumb rubber chemistry. It seems that higher CRM content has significant effect on the elastic recovery of the modified bitumen by increasing the rubber mass due to the absorption of maltense from the bitumen binder. Thus, the modified binder become more elastic and thus improved its resistance to elastic deformation under high tensile stress [1–3].

### 4.3. Wheel Tracking Test Results

**Figure 4:**
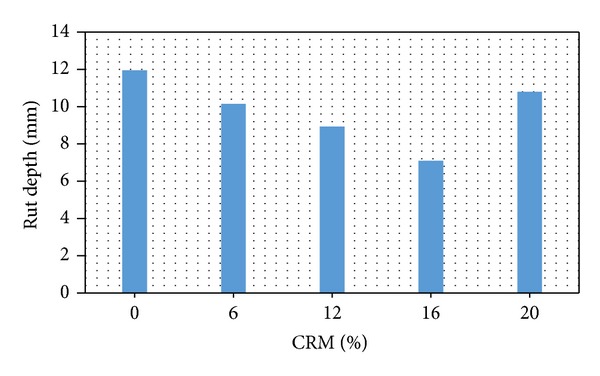
Rut depth versus CRM content.

Rutting is known as longitudinal depressions which follow the line of the wheel path. The wheel tracking test determines the susceptibility of bituminous mixtures to deform plastically at high temperatures under pressures similar to those experienced on the road. The susceptibility of bituminous to rutting is based on pass/failure criteria formed by repeated passes of a loaded wheel. This method measures the rut depth and number of pass to failure. The test measures the rutting of asphalt mixture by rolling a steel wheel across the surface of an asphalt mix slab that is held at temperature of 60°C [9]. The result obtained from wheel tracking test represented by rut depth for mixes with different CRM content at optimum binder content (OBC) is plotted in Figure 4. The figure shows that rut depth measurement provided a similar trend as compared to the creep test results, that is a reduction in rut depth as the crumb rubber content increases up to an optimum value which corresponds in the case to crumb rubber of 16%, and then it increases back with further inclusion of 20% CRM. The results could be explained by the physical and chemical interactions during the mixing process of the rubberised bitumen. Also, it could be attributed to the oily part of the bitumen being absorbed into the rubber powder and the increase in mass of the rubber particles, leading to the production of viscous gels that enables the bitumen binder to coat the aggregate effectively. Accordingly, an increase in binder mass could make the binder more elastic, stiff, and highly resistant to pavement rutting [1–3]. From Figure 4, the results show that the use of CRM in reinforcing SMA samples significantly improved the rutting performance as compared to the nonreinforced SMA (without CRM). The increase in rubber content leads to increase in elastic recovery results, and it is similar to ductility results of rubberised bitumen, which presented consistency with binder elasticity, rheology, rutting resistance, and elastic recovery after deformation. Hence, it leads to improving modified binder's resistance to rutting [1].

### 4.4. Relationship between Rutting Performance and Resilient Modulus and Creep Properties of CRM-Reinforced SMA Mix

**Figure 5:**
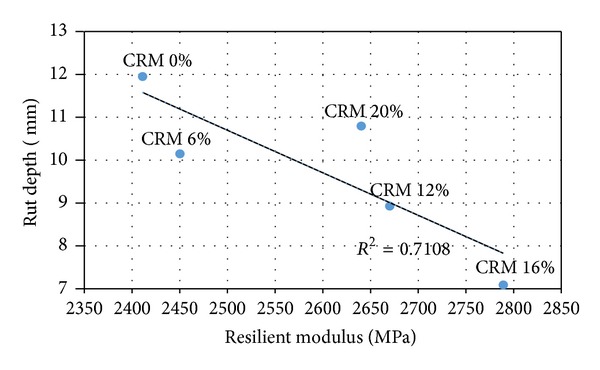
Rut depth versus resilient modulus (stiffness dynamic).

**Figure 6:**
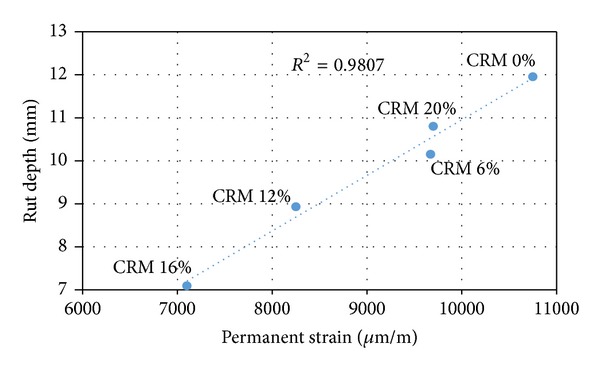
Rut depth versus creep permanent strain.

Figures 5 and 6 display the correlation between rut depth versus resilient modulus and the permanent strain, respectively. The figures showed an acceptable linear relationship between the rut resistance and the tested data (resilient modulus and permanent strain), due to the addition of CRM to ordinary SMA mixes. The correlation coefficient between the rutting resistance and resilient modulus (stiffness) is about 0.799, while a slightly higher correlation coefficient is about 0.89 between the rutting resistance and permanent strain (creep). The results indicated that the increase in stiffness properties of the samples due to the addition of CRM is followed by a decrease in rut depth as shown in Figure 5. Similarly an increase in permanent strain due to the addition of CRM of the samples is followed by an increase in rut depth as shown in Figure 6; from a scientific point of view this is understandable. According to the previous studies [7, 16], indirect tensile test (IDT) was used to identify and evaluate the performance of asphalt mixture; whereas dynamic creep test was deemed as a better alternative to be able to determine the creep deformation and quantify the permanent deformation of bituminous mixes. Further, the interpretation of the strain/time response of a material undergoing a creep test provides significant parameters, which describe the instantaneous elastic/plastic and viscoelastic/plastic components of the material response [7]. 

Permanent deformation or rutting takes place due to the complex stress-strain behaviour of the bituminous mix. Also, rut resistance is due to both cohesion and internal friction, where the significance of internal friction is contributing to better rut resistance; and mixes cohesion is the dominate factor in determining the rut resistance. By using the same concept resilient modulus and creep tests data can be related to cohesion and internal friction of asphalt and thus to rutting resistance. The resilient modulus test being a nondestructive test is in fact is a good indicator of mixture cohesion. On the other hand, creep test being a destructive test better accounts for internal friction [7]. In this study higher correlation coefficient was obtained between the rut depth and creep test as compared to resilient modulus; thus dynamic creep test might be a more reliable test in evaluating the rut resistance of asphalt mixture.

## 5. Conclusions

Based on test results the mixtures containing CRM resulted in higher resilient modulus, higher resistance to permanent strain, and higher resistance to rutting. It was obvious that the CRM content of 16% by weight of the total mix resulted in highest performance in terms of stiffness and resistance to permanent deformation as compared to the ordinary mix. Results indicated that CRM might enhance the rutting resistance of SMA mixture depending on its proportion. Rutting decreased as the crumb rubber content increases up to an optimum values then it increases back. As identifiable correlation between the laboratory tests parameters (resilient modulus and permanent strain) and rutting performance of CRM-reinforced-SMA mixture was obtained. The factor that probably influence the rutting performance could be attributed to the internal friction between the aggregate and mix cohesion. The dynamic creep test was found to be more reliable in evaluating the rutting resistance of asphalt. In addition the contribution of aggregates internal friction to the rut was found to be more significant than mix cohesion.

## Figures and Tables

**Table 1:** Properties of base binder grade 80/100 penetration.

Test properties	Test result
Viscosity @ 135°C (pas)	0.65
G*/sin *δ* @ 64°C (kPa)	1.35
Ductility @ 25°C	100
Softening point @ 25°C	47
Penetration @ 25°C	88

**Table 2:** Sieve analysis of tyre rubber.

Sieve no. (size, mm)	Particle size distribution % passing
No. 10 (2.00)	100
No. 16 (1.180)	100
No. 20 (0.850)	100
No. 30 (0.600)	97.7
No. 40 (0.425)	61.1
No. 50 (0.300)	33.9
No. 80 (0.180)	12.5
No. 100 (0.150)	7.5
No. 200 (0.075)	0.0

**Table 3:** SMA 20 aggregate gradation [9].

SMA 20					
BS Sieve	% Passing			% Retained	Weight (G)
	Min.	Max.	Mid.		
19	100	100	100	0	0
12.5	85	95	90	10	110
9.5	65	75	70	20	220
4.75	20	28	24	46	506
2.36	16	24	20	4	44
0.6	12	16	14	6	66
0.3	12	15	13.5	0.5	5.5
0.075	8	10	9	4.5	49.5
pan	0	0	0	9	99
				**100**	**1100**
